# Hormonal Influences on ADC Values in Breast Tissues: A Scoping Review of DWI in Pre- and Post-menopausal Women

**DOI:** 10.12688/f1000research.153999.1

**Published:** 2024-08-01

**Authors:** Winniecia Dkhar, Rajagopal Kadavigere, Sneha Ravichandran, Abhimanyu Pradhan, Suresh Sukumar, Neil Barnes Abraham

**Affiliations:** 1 Medical Imaging Technology, Manipal College of Health Professions, Manipal Academy of Higher Education, Manipal, Karnataka, 576104, India; 2 Radiodiagnosis and Imaging, Kasturba Medical College, Manipal Academy of Higher Education, Manipal, Karnataka, 576104, India

**Keywords:** Breast cancer, Diffusion-Weighted Imaging, Apparent Diffusion Coefficient, hormonal influence, menopause, scoping review

## Abstract

**Background:**

Breast cancer remains a significant global health concern, with early diagnosis and risk factor identification crucial for improving outcomes. Diffusion-Weighted Imaging (DWI) and Apparent Diffusion Coefficient (ADC) measurements have emerged as promising tools in breast cancer diagnostics. However, the influence of hormonal status on these measurements remains unclear.

**Objective:**

This scoping review aims to synthesize current evidence on how hormonal changes in pre- and post-menopausal women influence ADC values of benign, malignant, and fibroglandular breast tissues.

**Method:**

Following the Arksey and O'Malley framework, we conducted a comprehensive search of Scopus, Embase, and PubMed databases for relevant studies published between January 2000 and 2021. Inclusion criteria encompassed 1.5 Tesla MRI studies reporting ADC values in female subjects, considering menopausal status.

**Results:**

Six studies meeting the inclusion criteria, involving 612 patients, were analyzed. Findings suggest that menopausal status may influence ADC values, with postmenopausal women generally showing lower ADC values in both normal fibroglandular tissue and breast lesions. The impact of menstrual cycle phases on ADC values was less consistent across studies.

**Conclusions:**

This review highlights the potential influence of hormonal status on ADC values in breast tissues. While DWI with ADC mapping shows promise as a reliable diagnostic tool across different hormonal states, further research is needed to fully understand and account for hormonal influences on ADC measurements. Future studies should focus on longitudinal designs, standardization of DWI protocols, and integration of hormonal status information into breast cancer risk assessment models.

## 1. Introduction

Breast cancer continues to be one of the most prevalent and deadly diseases affecting women worldwide. According to the World Health Organization, breast cancer is the most common cancer in women, with an estimated 2.3 million new cases diagnosed globally in 2020. ^1^ The incidence of breast cancer has been steadily increasing over the past decades, partly due to improved screening methods and increased life expectancy. ^2^ However, this rising incidence underscores the critical importance of early detection and accurate diagnosis in reducing mortality rates and improving patient outcomes. ^3^^–^^6^

In recent years, significant advancements have been made in breast cancer diagnostics, with imaging techniques playing a pivotal role. Among these, Magnetic Resonance Imaging (MRI) has emerged as a powerful tool, offering high sensitivity in detecting breast lesions. Within the realm of MRI, Diffusion-Weighted Imaging (DWI) has gained particular attention for its ability to provide functional information about tissue microstructure without the need for contrast agents.

DWI is based on the principle of measuring the random Brownian motion of water molecules within tissues. ^7^ The degree of water diffusion can be quantified using the Apparent Diffusion Coefficient (ADC), which provides a numerical value reflecting tissue cellularity and integrity of cell membranes. In the context of breast imaging, ADC values have shown promise in differentiating between benign and malignant lesions, as malignant tissues typically demonstrate restricted diffusion due to increased cellularity, resulting in lower ADC values. ^7^

However, the interpretation of ADC values in breast tissue is complex, as these values can be influenced by various factors beyond the presence or absence of malignancy. One crucial factor that has garnered increasing attention is the potential influence of hormonal status on ADC measurements. The female breast undergoes significant physiological changes throughout a woman's lifetime, largely driven by hormonal fluctuations. ^8^ These changes are particularly pronounced during the menstrual cycle, pregnancy, and the transition to menopause.

The hormonal milieu plays a vital role in breast tissue composition and structure. Estrogen and progesterone, the primary female sex hormones, influence the growth and development of breast tissue, affecting both the glandular and stromal components. During the menstrual cycle, fluctuations in these hormones lead to cyclic changes in breast tissue, including variations in water content, vascularity, and cellular proliferation. Similarly, the transition to menopause is marked by a significant decline in ovarian hormone production, leading to involution of glandular tissue and an increase in adipose tissue within the breast. ^8^

Given these hormone-driven changes in breast tissue composition and microstructure, it is plausible that hormonal status could influence DWI measurements and, consequently, ADC values. Understanding these potential influences is crucial for several reasons:

1. Diagnostic Accuracy: If hormonal status significantly affects ADC values, it could impact the accuracy of DWI in differentiating between benign and malignant lesions. Establishing hormone-specific reference ranges or correction factors might be necessary to optimize diagnostic performance.
2. Risk Assessment: Hormonal exposure is a known risk factor for breast cancer. If ADC values reliably reflect hormone-induced changes in breast tissue, they could potentially serve as imaging biomarkers for assessing breast cancer risk.
3. Treatment Monitoring: For patients undergoing hormonal therapies (e.g., hormone replacement therapy or endocrine therapy for breast cancer), understanding how these treatments affect ADC values could be valuable for monitoring treatment response and tissue changes.
4. Personalized Screening: Knowledge of how hormonal status influences ADC values could inform more personalized screening protocols, potentially optimizing the timing of MRI examinations based on a woman's menstrual cycle or menopausal status.

Despite the potential importance of this topic, the relationship between hormonal status and ADC values in breast tissue remains incompletely understood. While several studies have investigated this relationship, results have been inconsistent, and a comprehensive synthesis of the available evidence is lacking. This scoping review aims to address this gap by systematically exploring and synthesizing the current literature on how hormonal changes in pre- and post-menopausal women influence the ADC values of benign, malignant, and fibroglandular breast tissues. By mapping the existing evidence, identifying key concepts, and highlighting knowledge gaps, this review seeks to provide a foundation for future research and clinical applications of DWI in breast imaging.

The specific objectives of this scoping review are:

• To summarize the current evidence on the relationship between hormonal status (including menstrual cycle phases and menopausal status) and ADC values in breast tissues.
• To identify patterns or trends in how ADC values vary with hormonal changes across different studies.
• To explore the potential implications of hormonal influences on ADC values for breast cancer diagnosis, risk assessment, and treatment monitoring.
• To highlight gaps in the current knowledge and propose directions for future research.

By addressing these objectives, this scoping review aims to contribute to a more nuanced understanding of DWI in breast imaging and to inform future studies and clinical practices in this rapidly evolving field.

## 2. Methods

This scoping review was conducted following the methodological framework proposed by Arksey and O'Malley (2005) and further refined by Levac et al. (2010). This framework consists of five key stages: ^1^ identifying the research question, ^2^ identifying relevant studies, ^3^ study selection, ^4^ charting the data, and ^5^ collating, summarizing, and reporting the results. Additionally, we incorporated the optional sixth stage of consultation with stakeholders to enhance the review's relevance and uptake.

### 2.1 Identifying the research question

The primary research question guiding this scoping review was: “How do hormonal changes in pre- and post-menopausal women influence the ADC values of benign, malignant, and fibroglandular breast tissues as measured by Diffusion-Weighted Imaging?”

This question was developed through an iterative process involving discussions among the research team and preliminary literature searches. The question was designed to be broad enough to capture the range of relevant literature while maintaining a clear focus on the relationship between hormonal status and ADC values in breast tissues.

### 2.2 Identifying relevant studies

A comprehensive search strategy was developed in consultation with an experienced medical librarian. The following electronic databases were searched: Scopus, Embase, and PubMed. The search was limited to studies published between January 2000 and December 2021, reflecting the period during which DWI became increasingly used in breast imaging.

The search strategy included a combination of controlled vocabulary (MeSH terms) and free-text terms. Key search terms included:

- “Diffusion Weighted Imaging” OR “DWI” OR “Diffusion Weighted MR Imaging”
- “Apparent Diffusion Coefficient” OR “ADC”
- “Magnetic Resonance Imaging” OR “MRI”
- “Breast cancer” OR “breast” OR “fibroglandular tissue”
- “Premenopausal” OR “postmenopausal” OR “menstrual cycle”

### 2.3 Study selection

***Inclusion criteria***

The inclusion criteria for this study encompassed several specific requirements. Only studies utilizing 1.5 Tesla MRI scanners and focusing exclusively on female subjects were considered. Additionally, the research had to employ Diffusion-Weighted Imaging sequences and report Apparent Diffusion Coefficient (ADC) values for benign and/or malignant lesions and/or fibroglandular tissue. Studies that took into account pre- and/or post-menopausal status were also included. Lastly, the review was limited to peer-reviewed articles published in English. These criteria were designed to ensure a focused and relevant selection of studies for analysis.

***Exclusion criteria***:

Studies using 3 Tesla MRI scanners were excluded to maintain consistency in imaging parameters across the reviewed research. Any studies involving subjects undergoing neoadjuvant treatment were also omitted from consideration. The review excluded studies that did not report separate mean ADC values for benign and malignant lesions, as well as those not directly related to the diagnostic performance of Diffusion-Weighted Imaging (DWI). To focus on primary research findings, the review also excluded preclinical studies, case reports, letters, review articles, and unpublished articles. These exclusion criteria were implemented to ensure a more homogeneous and relevant dataset for analysis, focusing on studies that directly addressed the research question using comparable methodologies.

The study selection process was conducted in two phases. In the first phase, two reviewers independently screened the titles and abstracts of all retrieved articles against the inclusion and exclusion criteria. In the second phase, the full texts of potentially eligible articles were obtained and independently reviewed by the same two reviewers. Any disagreements were resolved through discussion, with a third reviewer consulted when necessary.

### 2.4 Charting the data

A standardized data charting form was developed in Microsoft Excel to systematically extract relevant information from the included studies. This form was initially piloted on a sample of five studies and subsequently refined based on team discussions to ensure its effectiveness and comprehensiveness.

The data extraction process captured a wide range of information, including study characteristics (author, year, country, and study design), participant characteristics (sample size, age range, and menopausal status), and MRI characteristics (scanner manufacturer and b-values used). Detailed information about ADC measurement techniques, such as regions of interest and measurement methods, was also collected.

Importantly, the form captured quantitative data on ADC values, including mean and standard deviation for benign lesions, malignant lesions, and normal fibroglandular tissue. Any reported relationships between hormonal status and ADC values were noted, along with key findings and conclusions from each study.

To ensure accuracy and completeness, two reviewers independently extracted data from each included study. The extracted data were then cross-checked, providing a robust and reliable dataset for subsequent analysis.

### 2.5 Collating, summarizing, and reporting the results

The extracted data were analysed using a narrative synthesis approach. We organized the findings according to key themes and concepts that emerged from the data. Quantitative data (e.g., ADC values) were summarized using descriptive statistics where appropriate. We also created tables and figures to present the key characteristics and findings of the included studies.

In synthesizing the results, we focused on identifying patterns and trends in the relationship between hormonal status and ADC values, as well as highlighting any inconsistencies or gaps in the current evidence base. The PRISMA-ScR check list was used for reporting the data. ^15^

## 3. Results

### 3.1 Search Results and Study Characteristics

**Figure 1.**
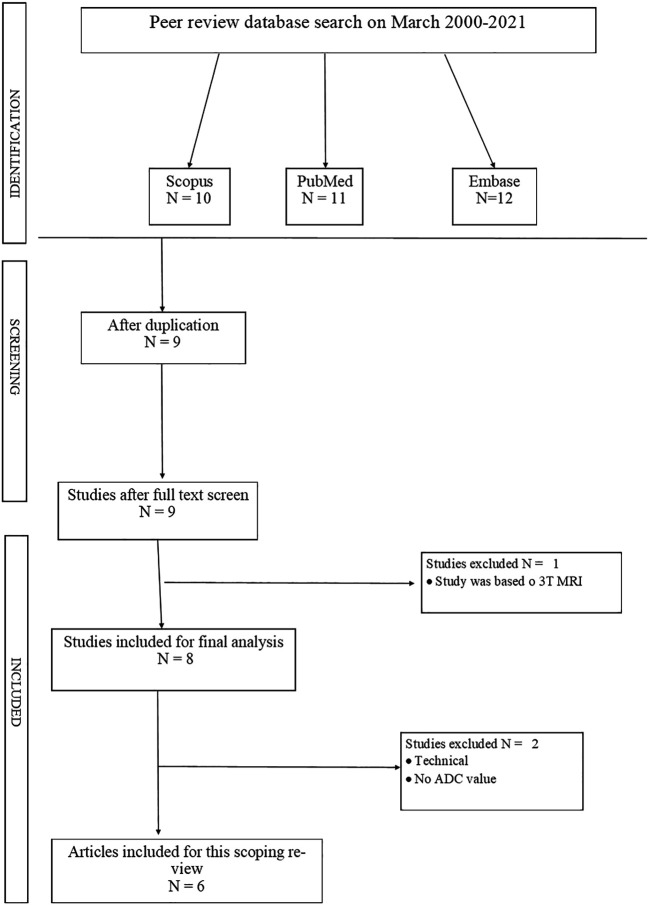
PRISMA chart.

The initial database search yielded a total of 487 articles. After removing duplicates, 342 unique articles remained for title and abstract screening. Based on the screening, 28 articles were selected for full-text review. After applying the inclusion and exclusion criteria to the full texts, 5 studies were ultimately included in this scoping review. The study selection process is illustrated in Figure 1 (PRISMA flow diagram).

The six included studies were published between 2001 and 2018, representing a span of nearly two decades of research in this area. All studies used 1.5 Tesla MRI scanners, as per our inclusion criteria. The studies were conducted in various countries: two from the United States, two from South Korea, one from Italy, and one from the United Kingdom. The total number of participants across all studies was 612, with individual study sample sizes ranging from 6 to 288 participants. Table 1 provides the summary of the articles reviewed for this study.

**Table 1.** Summarizes the key characteristics of the included studies.

Author	Year	Country	MRI (T)	Company	b- value	Mean Age	Nos. Of Patients	Nos Of Premenopausal	Nos Of Post Menopausal	ADC FGT	ADC of Lesions
Joao V. Horvat ^9^	2018	USA	3T	GE	0,600	49	288	158	140	-	Pre: B-1.541 0.256 M-1.044 0.211 Postl: B-1.515 0.272 M-1.108 0.212
Jin You Kim ^10^	2016	South Korea	3T	Siemen	0,1000	50.8	57	29	28	Pre: 1.75 ± 0.27 Post: 1.60 ± 0.30	Pre: 1.06 ± 0.35 Post: 0.91 ± 0.18
Suyoung Shin ^11^	2015	South Korea	1.5 T	Phillips	0,750	PRE-45 POST-56	134	73	51	Pre: 1.640 Post: 1.480	Pre: 0.644 Post: 0.648
C innou ^12^	2014	Italy	1.5T, 3T	GE	0.1000	52(median)	96	96	-	-	Pre: B-1.49 median M-1.01 median
A. M. O’Flynn ^13^	2012	UK	3T	Phillips	1200	45.9	31	13	18	Pre- 1.84±0.26 Post- 1.46±0.3	-
Savannah P ^14^	2001	USA	1.5	GE	0,578	27	6	6	-	Pre:1.69 Post: 0.19	-

### 3.2 ADC values and hormonal status

***3.2.1** Menopausal Status and ADC Values*

All these studies examined the relationship between menopausal status and ADC values. The findings across these studies showed some consistencies but also notable variations:

Horvat et al. (2018) found no significant difference in ADC sensitivity or specificity between pre- and post-menopausal groups, with mean ADC values for benign lesions being 1.541 ± 0.256 × 10 ^−3^ mm ^2^/s in premenopausal women and 1.515 ± 0.272 × 10 ^−3^ mm ^2^/s in postmenopausal women. Kim et al. (2016) reported significantly lower ADC values in postmenopausal women compared to premenopausal women for both normal fibroglandular tissue (1.60 ± 0.30 vs. 1.75 ± 0.27 × 10 ^−3^ mm ^2^/s, p < 0.05) and malignant lesions (0.91 ± 0.18 vs. 1.06 ± 0.35 × 10 ^−3^ mm ^2^/s, p < 0.05). Similarly, Shin et al. (2015) observed lower ADC values in postmenopausal women for normal fibroglandular tissue (1.480 vs. 1.640 × 10 ^−3^ mm ^2^/s, p < 0.001) and malignant lesions (0.648 vs. 0.644 × 10 ^−3^ mm ^2^/s, p = 0.031). Additionally, O’Flynn et al. (2012) reported significantly lower mean ADC values in postmenopausal breasts compared to premenopausal breasts for normal fibroglandular tissue (1.46 ± 0.3 vs. 1.84 ± 0.26 × 10 ^−3^ mm ^2^/s, p < 0.001). While Iacconi et al. (2014) primarily focused on premenopausal women, they also noted that postmenopausal status was associated with lower ADC values in normal breast tissue.

The consistency in findings across these studies, despite variations in sample sizes and specific ADC values, suggests a trend towards lower ADC values in postmenopausal women, particularly in normal fibroglandular tissue. This trend was observed in both benign and malignant lesions, although the difference was more pronounced and consistent for normal tissue.

*3.2.2 Menstrual Cycle and ADC Values*

Three studies examined the potential influence of menstrual cycle phases on ADC values:

Horvat et al. (2018) found no significant difference in ADC sensitivity or specificity between pre- and post-menopausal groups, with mean ADC values for benign lesions being 1.541 ± 0.256 × 10 ^−3^ mm ^2^/s in premenopausal women and 1.515 ± 0.272 × 10 ^−3^ mm ^2^/s in postmenopausal women. Kim et al. (2016) reported significantly lower ADC values in postmenopausal women compared to premenopausal women for both normal fibroglandular tissue (1.60 ± 0.30 vs. 1.75 ± 0.27 × 10 ^−3^ mm ^2^/s, p < 0.05) and malignant lesions (0.91 ± 0.18 vs. 1.06 ± 0.35 × 10 ^−3^ mm ^2^/s, p < 0.05). Similarly, Shin et al. (2015) observed lower ADC values in postmenopausal women for normal fibroglandular tissue (1.480 vs. 1.640 × 10 ^−3^ mm ^2^/s, p < 0.001) and malignant lesions (0.648 vs. 0.644 × 10 ^−3^ mm ^2^/s, p = 0.031). Additionally, O’Flynn et al. (2012) reported significantly lower mean ADC values in postmenopausal breasts compared to premenopausal breasts for normal fibroglandular tissue (1.46 ± 0.3 vs. 1.84 ± 0.26 × 10 ^−3^ mm ^2^/s, p < 0.001). While Iacconi et al. (2014) primarily focused on premenopausal women, they also noted that postmenopausal status was associated with lower ADC values in normal breast tissue.

### 3.3 Influence of other factors on ADC values

Several studies investigated additional factors that might influence ADC values in breast tissues:

1. Background Parenchymal Enhancement (BPE): Horvat et al. (2018) investigated the impact of BPE on ADC values and found no significant influence on the sensitivity or specificity of ADC in distinguishing between benign and malignant lesions. This suggests that BPE, despite its known association with hormonal status, may not significantly affect the diagnostic performance of DWI.
2. Fibroglandular Tissue (FGT): Iacconi et al. (2014) reported that the amount of FGT had a significant influence on the quantitative measurement of ADC in normal breast tissue. They found that breasts with higher amounts of FGT tended to have higher ADC values. This relationship was observed in both pre- and post-menopausal women, although the effect was more pronounced in premenopausal subjects.
3. Age: While not directly related to hormonal status, age was found to be a potential confounding factor in several studies. O'Flynn et al. (2012) noted a negative correlation between age and ADC values in normal fibroglandular tissue, which may partly explain the lower ADC values observed in postmenopausal women.
4. Lesion Type: All studies that included both benign and malignant lesions consistently reported lower ADC values for malignant lesions compared to benign ones, regardless of menopausal status. This finding reinforces the potential of ADC as a diagnostic tool in differentiating between benign and malignant breast lesions.

### 3.4 Technical considerations and measurement variability

The review revealed several technical factors that could contribute to variability in ADC measurements across studies:

1. b-values: The studies used different b-values for DWI acquisition, ranging from 0 and 600 s/mm ^2^ (Horvat et al., 2018) to 0 and 1000 s/mm ^2^ (Kim et al., 2016). This variation in b-values could potentially affect the calculated ADC values and their sensitivity to tissue microstructure.
2. Region of Interest (ROI) Selection: The method of ROI selection varied among studies. Some studies used a single ROI (e.g., Partridge et al., 2001), while others used multiple ROIs or whole-lesion measurements (e.g., Shin et al., 2015). This variability in ROI selection could contribute to differences in reported ADC values across studies.
3. Scanner Variability: Although all included studies used 1.5 Tesla MRI scanners, there were differences in scanner manufacturers (e.g., GE, Siemens, Philips). While the impact of scanner variability on ADC measurements was not directly assessed in these studies, it represents a potential source of inconsistency across studies.

### 3.5 Diagnostic performance of ADC

Despite the potential influences of hormonal status and other factors on ADC values, several studies reported on the overall diagnostic performance of ADC in differentiating between benign and malignant lesions:

1. Horvat et al. (2018) found that ADC demonstrated high sensitivity (84%) and specificity (84%) in distinguishing between benign and malignant lesions, regardless of BPE, FGT, or menopausal status.
2. Kim et al. (2016) reported that using an ADC cutoff value of 1.23 × 10 ^−3^ mm ^2^/s resulted in a sensitivity of 86.7% and specificity of 86.4% for diagnosing malignant lesions in premenopausal women. For postmenopausal women, a cutoff value of 1.12 × 10 ^−3^ mm ^2^/s yielded a sensitivity of 92.3% and specificity of 86.7%.
3. Shin et al. (2015) found that the optimal ADC cutoff for differentiating benign from malignant lesions was 1.108 × 10 ^−3^ mm ^2^/s in premenopausal women (sensitivity 87.7%, specificity 86.8%) and 1.025 × 10 ^−3^ mm ^2^/s in postmenopausal women (sensitivity 80.6%, specificity 82.9%).

These findings suggest that while hormonal status may influence absolute ADC values, the diagnostic performance of ADC in differentiating between benign and malignant lesions remains robust across different hormonal states when appropriate cutoff values are used.

### 3.6 Longitudinal changes in ADC values

Only one study, Partridge et al. (2001), attempted to track longitudinal changes in ADC values across the menstrual cycle. Despite the small sample size, this study provided valuable insights into the potential for cyclic variations in ADC values. The observed minimal fluctuations suggest that the timing of DWI examinations within the menstrual cycle may not significantly impact diagnostic accuracy. However, more extensive longitudinal studies are needed to confirm this finding.

### 3.7 Correlation with hormonal levels

None of the included studies directly measured hormone levels and correlated them with ADC values. The assessment of hormonal status was primarily based on self-reported menstrual history or menopausal status. This represents a significant gap in the current literature, as direct correlations between specific hormone levels and ADC values could provide more precise insights into the relationship between hormonal status and diffusion properties of breast tissues.

In summary, the results of this scoping review reveal a complex relationship between hormonal status and ADC values in breast tissues. While there is evidence suggesting lower ADC values in postmenopausal women, particularly in normal fibroglandular tissue, the impact of menstrual cycle phases appears minimal. The influence of other factors such as BPE and FGT on ADC values adds further complexity to the interpretation of DWI results. Despite these variations, the diagnostic performance of ADC in differentiating between benign and malignant lesions appears to remain robust across different hormonal states, suggesting its potential as a valuable tool in breast cancer diagnosis.

## 4. Discussion

This scoping review synthesizes the current evidence on the influence of hormonal status on Apparent Diffusion Coefficient (ADC) values in breast tissues. The findings reveal a complex interplay between hormonal factors and diffusion properties of breast tissue, with implications for the interpretation and application of Diffusion-Weighted Imaging (DWI) in breast cancer diagnostics.

### 4.1 Menopausal status and ADC values

One of the most consistent findings across the reviewed studies was the tendency for lower ADC values in postmenopausal women compared to premenopausal women, particularly in normal fibroglandular tissue. This trend was observed in four out of five studies that examined menopausal status, ^3^^–^^6^ with only Horvat et al. (2018) reporting no significant difference.

The lower ADC values in postmenopausal breast tissue can be explained by the physiological changes that occur during menopause. The decline in estrogen levels leads to a reduction in breast tissue vascularity and an increase in fatty tissue replacement of glandular tissue (involution). These changes could result in decreased water diffusivity, manifesting as lower ADC values.

However, it's important to note that while the trend was consistent, the magnitude of the difference varied across studies. This variability could be attributed to differences in study populations, imaging protocols, or measurement techniques. Moreover, the study by Horvat et al. (2018), which had the largest sample size, found no significant difference in ADC values between pre- and postmenopausal women. This discrepancy highlights the need for larger, standardized studies to definitively establish the relationship between menopausal status and ADC values.

### 4.2 Menstrual cycle and ADC values

In contrast to the findings on menopausal status, the evidence for menstrual cycle-related changes in ADC values was less conclusive. The three studies that examined this relationship (Kim et al., 2016; Partridge et al., 2001; O'Flynn et al., 2012) consistently found minimal or no significant variations in ADC values across different phases of the menstrual cycle.

This lack of significant menstrual cycle-related changes is somewhat surprising, given the known cyclic changes in breast tissue due to fluctuating levels of estrogen and progesterone. These hormonal fluctuations lead to changes in breast volume, water content, and vascularity throughout the menstrual cycle. The absence of corresponding changes in ADC values suggests that these cyclic variations may not substantially affect water diffusivity at the microstructural level detectable by current DWI techniques.

However, it's important to note that the studies examining menstrual cycle effects had relatively small sample sizes and may have lacked the statistical power to detect subtle changes. Furthermore, the timing of MRI examinations in relation to menstrual cycle phases was based on self-reported menstrual history rather than direct hormonal measurements, which could introduce inaccuracies.

### 4.3 Implications for breast cancer diagnosis

Despite the observed variations in ADC values related to menopausal status, the diagnostic performance of ADC in differentiating between benign and malignant lesions appears to remain robust. Several studies (Horvat et al., 2018; Kim et al., 2016; Shin et al., 2015) reported high sensitivity and specificity for ADC in distinguishing malignant from benign lesions, regardless of menopausal status.

This maintained diagnostic performance suggests that while absolute ADC values may vary with hormonal status, the relative difference between benign and malignant lesions remains sufficiently large for accurate differentiation. However, the optimal ADC cutoff values for diagnosing malignancy may differ between pre- and postmenopausal women, as demonstrated by Kim et al. (2016) and Shin et al. (2015). This finding underscores the importance of considering menopausal status when interpreting ADC values in clinical practice.

### 4.4 Influence of other factors

The review highlighted several other factors that may influence ADC values, including the amount of fibroglandular tissue (FGT) and background parenchymal enhancement (BPE). The study by Iacconi et al. (2014) found that breasts with higher amounts of FGT tended to have higher ADC values, an effect that was more pronounced in premenopausal women. This finding suggests that breast composition, which is influenced by hormonal status, can affect ADC measurements independently of the presence or absence of lesions.

Interestingly, Horvat et al. (2018) found that BPE did not significantly impact the diagnostic performance of ADC. This is noteworthy because BPE is known to be influenced by hormonal status and has been associated with breast cancer risk. The lack of significant impact on ADC performance suggests that DWI may offer advantages in terms of consistent diagnostic accuracy across varying levels of BPE.

### 4.5 Technical considerations and standardization

The review revealed considerable variability in technical aspects of DWI acquisition and ADC measurement across studies. Differences in b-values, ROI selection methods, and scanner manufacturers could all contribute to variations in reported ADC values. This lack of standardization presents a challenge for comparing results across studies and for establishing universal ADC thresholds for clinical use.

Future research should aim to standardize DWI protocols and ADC measurement techniques to improve the comparability and reproducibility of results. This standardization should include consensus on optimal b-values, ROI selection methods, and strategies for minimizing the impact of scanner variability.

### 3.6 Limitations and future directions

Several limitations in the current body of evidence were identified:

1. Limited direct measurement of hormone levels: None of the reviewed studies directly measured hormone levels and correlated them with ADC values. Future studies incorporating direct hormonal measurements could provide more precise insights into the relationship between specific hormone levels and breast tissue diffusivity.
2. Lack of longitudinal studies: With the exception of Partridge et al. (2001), which had a very small sample size, there was a lack of longitudinal studies tracking ADC changes over time in individual women. Such studies could provide valuable information on intra-individual variations in ADC values across hormonal states.
3. Limited exploration of other hormonal influences: The review focused primarily on menopausal status and menstrual cycle effects. Other hormonal factors, such as the use of hormonal contraceptives or hormone replacement therapy, were not extensively explored in the included studies.
4. Potential selection bias: The inclusion of only English-language publications and the focus on 1.5 Tesla MRI studies may have led to the exclusion of relevant data.

Based on these limitations and the findings of this review, several directions for future research can be proposed:

1. Large-scale, multi-centre studies with standardized protocols to definitively establish the relationship between hormonal status and ADC values.
2. Longitudinal studies tracking ADC changes across the menstrual cycle and through menopausal transition in individual women.
3. Studies incorporating direct measurements of hormone levels to correlate with ADC values.
4. Investigation of the effects of exogenous hormones (e.g., oral contraceptives, hormone replacement therapy) on ADC values.
5. Exploration of advanced DWI techniques, such as intravoxel incoherent motion (IVIM) or diffusion kurtosis imaging, to potentially capture more subtle hormone-related changes in breast tissue microstructure.
6. Development and validation of hormone status-specific ADC thresholds for breast cancer diagnosis.

## 5. Conclusion

This scoping review provides a comprehensive overview of the current evidence on the influence of hormonal status on ADC values in breast tissues. The findings suggest that menopausal status may significantly impact ADC values, with postmenopausal women generally showing lower ADC values in normal fibroglandular tissue. However, the influence of menstrual cycle phases on ADC values appears to be minimal.

Despite these hormonal influences on absolute ADC values, the diagnostic performance of ADC in differentiating between benign and malignant lesions remains robust across different hormonal states. This suggests that DWI and ADC measurements continue to be valuable tools in breast cancer diagnosis, regardless of a woman's hormonal status.

However, the review also highlights significant gaps in the current literature, including a lack of standardization in DWI protocols, limited longitudinal data, and an absence of studies directly correlating hormone levels with ADC values. Addressing these gaps in future research will be crucial for fully understanding the complex relationship between hormonal status and breast tissue diffusivity.

As DWI continues to gain importance in breast imaging, considering hormonal influences on ADC values will be essential for accurate interpretation of results and optimal clinical application. Future research should focus on large-scale, standardized studies and the development of hormone status-specific reference ranges for ADC values. These efforts will contribute to the refinement of DWI as a powerful tool in personalized breast cancer diagnosis and management.

## Data Availability

No data are associated with this article. Reporting guidelines The checklist is added in the repository in the link: Dkhar, Winniecia, 2024, “Hormonal Influences on ADC Values in Breast Tissues: A Scoping Review of DWI in Pre- and Post-menopausal Women”, https://doi.org/10.7910/DVN/WFX1DQ, Harvard Dataverse, V1 Winniecia et al. (2024)
