# Supplementary material for: Hormonal Influences on ADC Values in Breast Tissues: A Scoping Review of DWI in Pre- and Post-menopausal Women
Source: F1000Res. 2025 Jan 27;13:857. Originally published 2024 Aug 1. [Version 3] doi: 10.12688/f1000research.153999.3 (PMC11783040; doi:10.12688/f1000research.153999.3)
Supplement: Peer review history

# Reviewer report: Reviewer response for version 3

**DOI:** 10.5256/f1000research.176915.r362771

**Published:** 2025-01-29

**Authors:** Avantsa Rohini

**Affiliations:** 1 Department of Radiodiagnosis and Imaging, MNR Medical College, Sangareddy, Telangana, India

The suggestions from the previous version were incorporated in the current version. I have no further comments to make.

Are the rationale for, and objectives of, the Systematic Review clearly stated?

Yes

Is the statistical analysis and its interpretation appropriate?

Yes

If this is a Living Systematic Review, is the ‘living’ method appropriate and is the search schedule clearly defined and justified? (‘Living Systematic Review’ or a variation of this term should be included in the title.)

Yes

Are sufficient details of the methods and analysis provided to allow replication by others?

Yes

Are the conclusions drawn adequately supported by the results presented in the review?

Yes

Reviewer Expertise:

breast imaging and  onco imaging

I confirm that I have read this submission and believe that I have an appropriate level of expertise to confirm that it is of an acceptable scientific standard.

# Reviewer report: Reviewer response for version 3

**DOI:** 10.5256/f1000research.176915.r362772

**Published:** 2025-01-28

**Authors:** Manna Debnath

**Affiliations:** 1 Charotar University of Science and Technology, Changa, Gujarat, India

Approved.

Are the rationale for, and objectives of, the Systematic Review clearly stated?

Yes

Is the statistical analysis and its interpretation appropriate?

Yes

If this is a Living Systematic Review, is the ‘living’ method appropriate and is the search schedule clearly defined and justified? (‘Living Systematic Review’ or a variation of this term should be included in the title.)

Yes

Are sufficient details of the methods and analysis provided to allow replication by others?

Yes

Are the conclusions drawn adequately supported by the results presented in the review?

Yes

Reviewer Expertise:

Expert in Medical Imaging Technology (MRI)

I confirm that I have read this submission and believe that I have an appropriate level of expertise to confirm that it is of an acceptable scientific standard.

# Reviewer report: Reviewer response for version 2

**DOI:** 10.5256/f1000research.170311.r355176

**Published:** 2025-01-08

**Authors:** Avantsa Rohini

**Affiliations:** 1 Department of Radiodiagnosis and Imaging, MNR Medical College, Sangareddy, Telangana, India

This is a very well written and interesting scoping review and the authors had done an excellent work. I think this contributes towards further research/alternatives. There are few suggestions the author needs to look into which might add further to the quality of the manuscript.

1. The introduction part of the manuscript explained well explained the need of the study and the highlighted the lacunae in the research. The following statements I would want author to reconsider.
2. Introduction First paragraph: 4 ^th^ line-“increased breast cancer incidence is partly due to increased life expectancy”. I suggest changing it to “due to increased awareness of the screening programme”.
3. Introduction 4 ^th and 5th^ paragraph: 4 ^th^ line –“hormonal fluctuations”-instead change to “hormonal phases” as fluctuations are abnormal phenomenon.
4. The study methodology is well written and the selection criteria were clear and correctly identified.
5. In the PRISMA flow chart, I suggest author to relook into this- ‘Study excluded N = 1 *Study was based on 3T MRI’, as this was supposed to be eliminated in the previous step.
6. The conclusion shows that the hypothesis was proven correctly.

Are the rationale for, and objectives of, the Systematic Review clearly stated?

Yes

Is the statistical analysis and its interpretation appropriate?

Yes

If this is a Living Systematic Review, is the ‘living’ method appropriate and is the search schedule clearly defined and justified? (‘Living Systematic Review’ or a variation of this term should be included in the title.)

Yes

Are sufficient details of the methods and analysis provided to allow replication by others?

Yes

Are the conclusions drawn adequately supported by the results presented in the review?

Yes

Reviewer Expertise:

breast imaging and  onco imaging

I confirm that I have read this submission and believe that I have an appropriate level of expertise to confirm that it is of an acceptable scientific standard.

# Reviewer report: Reviewer response for version 2

**DOI:** 10.5256/f1000research.170311.r319001

**Published:** 2024-09-13

**Authors:** Manna Debnath

**Affiliations:** 1 Charotar University of Science and Technology, Changa, Gujarat, India

I am satisfied with the changes authors have made to the manuscript. Author revisions have effectively addressed the concerns and suggestions raised during the review process.

Are the rationale for, and objectives of, the Systematic Review clearly stated?

Yes

Is the statistical analysis and its interpretation appropriate?

Yes

If this is a Living Systematic Review, is the ‘living’ method appropriate and is the search schedule clearly defined and justified? (‘Living Systematic Review’ or a variation of this term should be included in the title.)

Yes

Are sufficient details of the methods and analysis provided to allow replication by others?

Yes

Are the conclusions drawn adequately supported by the results presented in the review?

Yes

Reviewer Expertise:

Expert in Medical Imaging Technology (MRI)

I confirm that I have read this submission and believe that I have an appropriate level of expertise to confirm that it is of an acceptable scientific standard.

# Reviewer report: Reviewer response for version 1

**DOI:** 10.5256/f1000research.168971.r310441

**Published:** 2024-08-09

**Authors:** Manna Debnath

**Affiliations:** 1 Charotar University of Science and Technology, Changa, Gujarat, India

This is a scientifically sound scoping review. The author(s) have written extremely well. However, a few minor changes need to be addressed. Once these are included, the manuscript's quality will improve even further.

**Reviewers comment:**

 1. References are needed in the second paragraph of the introduction section.

 2. Page No.3 References are needed on the line ‘While several studies have investigated this relationship, results have been inconsistent, and a comprehensive synthesis of the available evidence is lacking.’

 3. The introduction is well-written and properly highlights the research gaps.

 4. In the study selection section, I suggest that the author include a statement on including or excluding full-text articles or abstracts.

 5. The phases of the selection process are well written.

 6. In the result section, paragraph 1, you stated that '5 studies were ultimately included for scoping review', yet in PRISMA flow diagram Figure 1, in the Included Section of the chart, the number of articles included for this scoping review is 6. Please confirm the mismatch between the included articles in the study.

 7. In the PRISMA flow chart, please correct the sentence, ‘Study excluded N = 1 *Study was based on 3T MRI.’

 8. Page no. 7, 3.2.1 Menopausal status and ADC values; and Page no. 8, 3.2.2 Mensural cycle and ADC values. Both paragraphs have the same data. Without repeating the data, I suggest you change the heading to Mensural cycle, menopausal status, and ADC values.

 9. Page 10, last paragraph: 'Future research'. I suggest fusing these lines on the next page, under the heading ‘Based on these limitations and the findings of this review, several directions for future research can be proposed’.

 10. The conclusion is clearly written and shows that the hypothesis was proven correctly.

Are the rationale for, and objectives of, the Systematic Review clearly stated?

Yes

Is the statistical analysis and its interpretation appropriate?

Yes

If this is a Living Systematic Review, is the ‘living’ method appropriate and is the search schedule clearly defined and justified? (‘Living Systematic Review’ or a variation of this term should be included in the title.)

Yes

Are sufficient details of the methods and analysis provided to allow replication by others?

Yes

Are the conclusions drawn adequately supported by the results presented in the review?

Yes

Reviewer Expertise:

Expert in Medical Imaging Technology (MRI)

I confirm that I have read this submission and believe that I have an appropriate level of expertise to confirm that it is of an acceptable scientific standard.

# Response

**Published:** 2024-08-12

**Authors:** Winniecia Dkhar

**Affiliations:** Medical Imaging Technology, Manipal Acdemy of Higher Education, Manipal, Karnataka, India

Thank you for your valuable feedback on our scoping review. We appreciate your thoughtful comments and suggestions. Here is our response to each of your points:

 1. We agreed and added appropriate references to the second paragraph of the introduction.

 2. We included relevant references to support the statement about inconsistent results and lack of comprehensive synthesis.

 3. Thank you for your positive feedback on the introduction.

 4. We added a clear statement about the inclusion/exclusion of full-text articles and abstracts in the study selection section.

 5. We appreciate your positive comment on the description of the selection process phases.

 6. Thank you for catching this discrepancy. We corrected the text to match the PRISMA flow diagram, confirming that 6 studies were included in the final review.

 7. We revised the sentence in the PRISMA flow chart as suggested.

 8. We appreciated this suggestion and merged the two sections under a single heading "Menstrual cycle, menopausal status, and ADC values" to avoid repetition.

 9. We had already implemented this suggestion by fusing the future research directions with the conclusion section.

 10. Thank you for your positive feedback on the conclusion.

 We made all these revisions to improve the quality and clarity of our manuscript. We appreciate your thorough review and constructive comments, which have undoubtedly enhanced the overall quality of our scoping review.
